# Staffing of Qualified Social Workers and Nursing Home Quality of Care

**DOI:** 10.1001/jamanetworkopen.2026.0074

**Published:** 2026-02-25

**Authors:** Yutong Chen, Ling Xu, Wei Jiang, Noelle Fields

**Affiliations:** 1 Department of Economics, University of Texas at Arlington; 2 School of Social Work, University of Texas at Arlington; 3 Department of Mathematics, Division of Data Science and Center of Innovation for Health Informatics, University of Texas at Arlington

## Abstract

**Importance:**

Nursing homes provide 24-hour care for more than 1.2 million US residents with complex medical and psychosocial needs. Social workers play a role in supporting person-centered care, yet many facilities lack adequate social worker coverage, and evidence associating qualified social worker staffing with quality outcomes remains limited.

**Objective:**

To examine the association between staffing of qualified social workers and nursing home quality outcomes, with attention to facilities serving residents with Alzheimer disease and related dementias.

**Design, Setting, and Participants:**

This facility-level cohort study used a synthetic difference-in-differences design with staggered adoption to analyze 2491 US nursing homes from January 1, 2017, to December 31, 2021, comparing facilities that employed a qualified social worker during 2018 to 2021 with those that had not yet employed one. Data analysis was conducted between October 1, 2024, and December 29, 2025.

**Exposure:**

Employment of a qualified social worker, defined as any facility with an annual mean of greater than 0 daily hours worked by employed (noncontract) qualified social workers.

**Main Outcomes and Measures:**

The primary outcome was the proportion of residents restrained. Secondary outcomes include hospitalizations per resident-year, 30-day rehospitalization among postacute admissions, and successful discharge to the community within 100 days.

**Results:**

The analysis included 2491 facilities. The mean (SD) resident age was 79.0 (5.9) years, and the mean (SD) share of female admissions was 0.58 (0.10). The mean (SD) Medicaid share was 0.61 (0.21), and the mean (SD) Medicare share was 0.14 (0.11). Qualified social worker staffing was associated with a 0.3–percentage point reduction in restraint use (95% CI, −0.6 to −0.0 percentage points), 46% of the 2017 mean. No significant associations were observed for hospitalizations, rehospitalization rates, or discharges. In subgroup analyses, facilities with above-median dementia prevalence had 0.5–percentage point lower restraint use (95% CI, −0.9 to –0.1 percentage points), with no association in low-prevalence facilities.

**Conclusions and Relevance:**

In this cohort study of US nursing homes, employment of a qualified social worker was associated with reduced restraint use, particularly in facilities with higher dementia prevalence. These findings support policies to expand social work capacity to promote person-centered, less-restrictive care in nursing homes.

## Key Points

**Question:**

What is the association between employment of qualified social workers and nursing home quality of care?

**Findings:**

In this cohort study using a synthetic difference-in-differences approach with staggered adoption, staffing of qualified social workers was associated with a statistically significant reduction in the use of restraints, with strong associations in facilities with a higher prevalence of Alzheimer disease and related dementias.

**Meaning:**

This study suggests that staffing of qualified social workers is associated with improved quality of care in nursing homes, particularly in facilities serving residents with cognitive impairment.

## Teaser

This cohort study examines the association between staffing of qualified social workers and nursing home quality of care, particularly in facilities serving residents with Alzheimer disease and related dementias.

## Introduction

Nursing homes are central to the US long-term care system, providing around-the-clock medical and personal support to approximately 1.2 million residents across 15 000 facilities.^1^ Despite the essential role of nursing homes, concerns about quality of care persist, including high hospitalization and rehospitalization rates, low successful discharge rates, and continued reliance on restrictive practices. Federal policy has long emphasized the importance of psychosocial services in nursing homes beyond medical care alone. The Omnibus Budget Reconciliation Act of 1987 requires Medicare- or Medicaid-certified facilities to provide medically related social services to support residents’ physical, mental, and psychosocial well-being. Under federal regulations, facilities with more than 120 beds must employ a qualified social worker (QSW) full time, whereas smaller facilities are exempt.^2^ A QSW generally holds a bachelor’s degree in social work or a related human services field and has supervised experience in health care settings.

QSWs play a critical role in nursing homes by addressing residents’ psychosocial needs, which are often as important as their medical conditions. Their responsibilities include assessment, care planning, psychosocial interventions, family counseling, care coordination, and advocacy for person-centered, less-restrictive environments.^3^ A recent report highlights the importance of QSWs on interdisciplinary care teams and summarizes evidence linking social work involvement with improved quality of care.^1^ Through these functions, QSWs may influence facility-level outcomes by managing residents’ behavioral symptoms, reducing reliance on restraints, and supporting care transitions through discharge planning and family communication—mechanisms aligned with federal quality monitoring and reimbursement priorities.^4,5^ Prior evidence, largely descriptive or qualitative, suggests associations between QSWs and improved resident health, behavioral, and service use outcomes,^6,7,8,9^ yet persistent workforce shortages and high turnover limit coverage, underscoring the need for more rigorous evidence.^10,11^

These challenges are especially pronounced in nursing homes serving residents with Alzheimer disease and related dementias (ADRD).^12,13^ Residents with cognitive impairment are at heightened risk of rehospitalization, restraint use, and other potentially harmful interventions, making person-centered care particularly urgent.^14,15,16^ Facilities with a higher ADRD prevalence often face greater staff burden and variable adherence to best practices, underscoring the need for targeted approaches.^17,18^ Evaluating the association between QSWs and outcomes in these settings is therefore important to assess whether they may mitigate risks and promote safer, individualized care.

Despite policy mandates and prior descriptive research, little is known about whether employing QSWs is associated with improved measurable quality outcomes. To fill this gap, we used national longitudinal data and a quasi-experimental difference-in-differences design to examine the association between QSW staffing and nursing home quality-of-care outcomes. We examined restraint use, hospitalizations, rehospitalizations, and successful discharges, with particular attention to facilities serving a higher proportion of residents with ADRD. This study extended prior work by providing, to our knowledge, the first quasi-experimental evidence at the national level on the association between QSW staffing and nursing home quality outcomes.

## Methods

This cohort study was approved by the University of Texas at Arlington institutional review board, which determined that the study was exempt and waived patient consent because it used publicly available, deidentified institution-level data. The study followed the Strengthening the Reporting of Observational Studies in Epidemiology (STROBE) reporting guideline.

### Data and Study Outcomes

We drew on 2 national data sources to construct facility-level measures of social work staffing, resident and facility characteristics, and care quality outcomes in US nursing homes from January 1, 2017, to December 31, 2021. The first was the Long-Term Care Focus (LTCFocus) database, developed by Brown University’s Center for Gerontology and Healthcare Research.^19^ LTCFocus provides annual information on Medicare- and Medicaid-certified nursing facilities in 48 continental states and Hawaii, excluding Alaska and Washington, DC, and includes data on resident demographics, facility ownership, staffing, and quality indicators. The second source was the Payroll-Based Journal (PBJ) nonnursing staff files, reported by Medicare- and Medicaid-certified nursing facilities to the Centers for Medicare & Medicaid Services (CMS). These files contained detailed information on daily staffing, including the variable Hrs_QualSocWrk_emp, which recorded the total daily hours worked by employed QSWs. The 2 datasets were linked using common facility identifiers and year, yielding a 96.3% facility-year match rate.

Our exposure of interest was the employment of a QSW. We measured QSW staffing as the annual mean daily hours worked by employed (noncontract) QSWs at each facility, calculated across all calendar days. A facility was classified as employing a QSW if this mean exceeded 0. In 2017, the mean (SD) number of employee QSW hours among facilities reporting any hours was 6.5 (5.0), and the median number of employee QSW hours was 5.2 hours (IQR, 4.3-7.4 hours), while 27.5% of facilities reported 0 mean daily employee QSW hours. We conducted a sensitivity analysis using a higher threshold of 1 or more mean daily hours to distinguish facilities with more regular staffing from those with minimal reported hours.

Our primary outcome was restraint use, derived by LTCFocus from CMS Certification and Survey Provider Enhanced Reporting (CASPER) inspection data. *Restraint use* was defined as the proportion of residents restrained at the annual certification survey. We selected restraint use as the primary outcome because it is a salient indicator of psychosocial and person-centered care, aligns closely with the professional responsibilities of QSWs, and is a key regulatory quality measure emphasized in federal nursing home oversight and policy.

We also examined secondary outcomes capturing broader dimensions of nursing home quality of care, all derived from Minimum Data Set assessment: (1) hospitalizations per resident-year, defined as the number of hospital transfers from the facility during the year divided by total resident-years; (2) 30-day rehospitalization rates, defined as the proportion of new postacute admissions from hospitals transferred to an acute hospital within 30 days; and (3) successful discharge rates, defined as the proportion of new postacute hospital admissions discharged alive to the community within 100 days. Because these outcomes rely on facility-reported data, measurement error could bias estimates in either direction^20,21^; if underreporting is nondifferential, estimated associations would be attenuated.

### Statistical Analysis

Statistical analysis was conducted between October 1, 2024, and December 29, 2025. We used a staggered adoption synthetic difference-in-differences (SDID) design to compare changes in quality-of-care outcomes in nursing homes before and after the employment of a QSW.^22,23^ This data-driven method combines features of traditional difference-in-differences and synthetic control approaches and has been increasingly used in health and policy evaluations.^24,25^ Like the difference-in-differences approach, the SDID approach estimates differential changes in outcomes before and after treatment; like synthetic control, it reweights control units to better match the pretreatment trends of treated units. The model incorporates facility fixed effects, which absorb time-invariant facility characteristics as well as time-invariant county- and state-level factors, and year fixed effects to capture common temporal shocks, including those related to the COVID-19 pandemic. We did not include additional covariates in the main specification.

The SDID estimator is obtained by solving a regularized least-squares problem that jointly estimates the mean treatment effect and data-driven unit and time weights. Conceptually, it constructs a synthetic comparison group by reweighting outcomes from facilities that had not yet employed a QSW in a given year—including facilities that never employed a QSW during 2017 to 2021—to closely mirror the pretreatment outcome dynamics of facilities that first employed a QSW between 2018 and 2021. By aligning treated and control facilities on pretreatment outcome trajectories while allowing baseline outcome levels to differ, the SDID approach helps mitigate concerns that QSW staffing may be nonrandomly adopted across nursing homes based on prior outcome trends. Additional technical details on staggered-adoption SDID implementation are provided in the eMethods in Supplement 1.

We reported bootstrap standard errors based on 500 repetitions. The bootstrap resamples facilities with replacement to approximate the sampling distribution. All statistical analyses were performed in Stata, version 19 (StataCorp LLC). All tests were 2-sided, and statistical significance was defined as *P* < .05.

The LTCFocus-PBJ dataset included 15 790 nursing homes observed between 2017 and 2021. To satisfy the balanced panel requirement of the SDID approach, we first restricted the sample to facilities with complete 5-year observations on all 4 outcome variables, the treatment variable, and a set of facility characteristics (Table 1); facilities with missing data were excluded. We then excluded facilities that had already employed a QSW in 2017, as these facilities had no untreated period from which to construct a valid counterfactual. We also excluded facilities that entered and exited QSW employment during the study period, as the SDID approach requires monotonic treatment adoption. After applying these restrictions, the final analytic sample consisted of 2491 nursing homes spanning 49 states and 174 counties. Among them, 1077 facilities employed a QSW between 2018 and 2021, while 1414 facilities never employed a QSW during the study period. The estimates in this study should thus be interpreted cautiously, as they captured the association between QSW staffing and outcomes among nursing homes without a QSW at baseline (2017).

**Table 1.** Summary Statistics of Facility Characteristics: Facilities Excluded vs Included in the Main Analysis, 2017

Variable	Excluded		Included		Difference^a^	*P* value
	No.	Mean (SD)	No.	Mean (SD)		
Total beds, No.	12 551	108.7 (64.0)	2491	100.4 (43.5)	8.3 (1.3)	<.001
Has Alzheimer disease SCU	12 552	0.2 (0.4)^b^	2491	0.1 (0.3)^b^	0.01 (0.01)^b^	.32
Has any SCU	12 552	0.2 (0.4)^b^	2491	0.2 (0.4)^b^	0.02 (0.01)^b^	.03
Chain affiliation	12 552	0.6 (0.5)^b^	2491	0.6 (0.5)^b^	−0.1 (0.01)^b^	<.001
For profit	12 552	0.7 (0.5)^b^	2491	0.8 (0.4)^b^	−0.1 (0.01)^b^	<.001
Hospital based	12 552	0.1 (0.2)^b^	2491	0.02 (0.1)^b^	0.04 (0.01)^b^	<.001
Age of residents, y	12 331	79.3 (7.5)	2491	79.0 (5.9)	0.3 (0.2)	.08
Medicaid share of residents	12 551	0.6 (0.2)	2491	0.6 (0.2)	−0.02 (0.01)	<.001
Medicare share of residents	12 551	0.1 (0.1)	2491	0.1 (0.1)	0.01 (0.003)	.07
RN hours per resident-day	11 031	0.5 (0.5)	2491	0.6 (0.3)	0.1 (0.01)	<.001
LPN hours per resident-day	11 031	0.8 (0.4)	2491	0.8 (0.3)	0.02 (0.01)	.01
CNA hours per resident-day	11 031	2.2 (0.6)	2491	2.2 (0.5)	0.1 (0.01)	<.001

Abbreviations: CNA, certified nursing assistants; LPN, licensed practical nurse; RN, registered nurse; SCU, special care unit.

^a^ The mean differences between facilities excluded from the main analysis and those included, along with corresponding standard errors from a 2-sample *t* test assuming equal variances.

^b^ Indicator variable equals 1 if a facility has a given trait and 0 otherwise.

## Results

### Summary Statistics

The primary analysis sample included 2491 nursing homes with a mean (SD) of 100.4 (43.5) beds, a mean resident age of 79.0 (5.9) years, a mean (SD) share of female admissions of 0.58 (0.10), a mean (SD) Medicaid share of 0.61 (0.21), and a mean (SD) Medicare share of 0.14 (0.11). Table 1 compares baseline characteristics of included facilities with those excluded because they had already employed a QSW in 2017. Excluded facilities were larger, less likely to be for-profit facilities or chain-affiliated facilities, and more likely to be hospital-based facilities. Included facilities had residents who were, on average, 0.3 years younger (95% CI, −0.03 to 0.6 years) and had a 1.8 percentage point (95% CI, −2.8 to −0.8 percentage points) higher Medicaid share. In terms of staffing, excluded facilities devoted more staff time per resident-day across registered nurses, licensed practical nurses, and certified nursing assistants. Overall, facilities that had already employed a QSW in 2017 were larger and more resource intensive. Therefore, restricting the sample to facilities without baseline QSWs might yield conservative estimates of the association between QSW staffing and nursing home outcomes.

We additionally presented summary statistics comparing included facilities with facilities that had complete 5-year data and did not employ a QSW in 2017 but entered and exited QSW staffing between 2017 and 2021 (eTable 1 in Supplement 1). A joint multivariate test indicated that these 2 groups were not statistically different at baseline (*F* = 0.63; *P* = .82). In addition, eTable 2 in Supplement 1 reports descriptive statistics for treated and control facilities in 2017 within the primary analytic sample, highlighting baseline differences (eg, total number of beds and chain affiliation) that motivated the use of the SDID approach.

### Graphical Analysis

**Figure 1.**
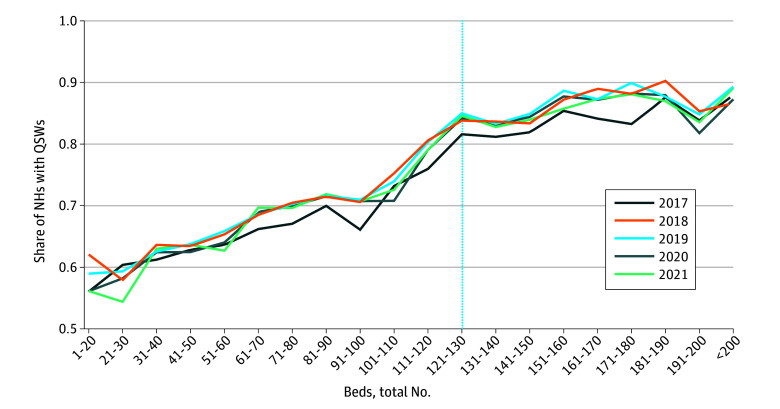
Share of Nursing Homes (NHs) With Qualified Social Workers (QSWs), by Bed Capacity and Year Data are drawn from the Long-Term Care Focus database and the Payroll-Based Journal daily nonnursing staff records to identify each facility’s bed capacity and the employment of a QSW. A facility is classified as employing a QSW if the annual mean of total daily hours worked by QSW staff exceeds 0. The figure is based on a sample of 15 790 facilities between 2017 and 2021.

Figure 1 plots the share of nursing homes employing a QSW by facility size, using data from 15 790 facilities observed between 2017 and 2021. Compliance with the federal requirement that facilities with more than 120 beds employ a QSW was incomplete. The likelihood of employing a QSW increased steadily with facility size rather than displaying a marked increase at the 120-bed threshold. Even among facilities above the 120-bed cutoff, a substantial share did not comply. For example, 16.2% of nursing homes (709 of 4380) with more than 120 beds lacked a QSW in 2017. This variation highlighted the uneven implementation of federal standards and motivated examining whether differences in QSW staffing were associated with resident outcomes using the SDID framework.

### Staggered SDID Estimates

**Figure 2.**
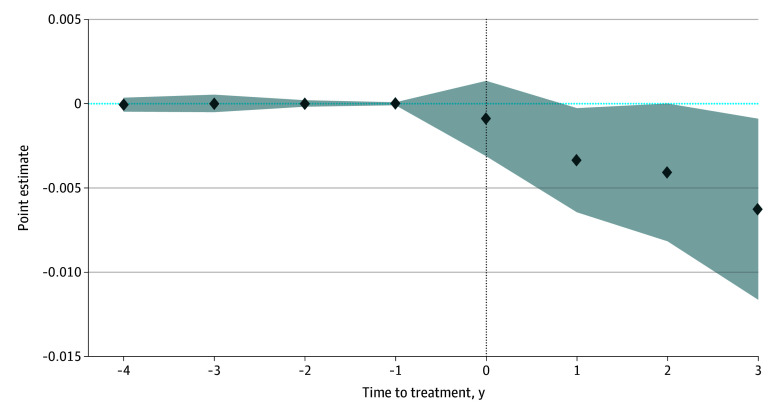
Event-Study Estimates of the Association Between Qualified Social Worker Employment and Resident Restraint Use, 2017-2021 The figure reports event-study estimates using the synthetic difference-in-differences method with staggered adoption. The analytic sample consists of 2491 nursing facilities observed annually from 2017 through 2021. The outcome variable is the proportion of residents restrained at the annual certification survey. The vertical dotted line at 0 corresponds to the year in which a facility first employed a qualified social worker. Time to treatment = −1 year serves as the reference (baseline) year. Shaded areas indicate 95% CIs. We conducted a joint *F* test of the pretreatment coefficients, which failed to reject the null hypothesis of no differential pretreatment trends at the 5% level (*P* = .99).

Table 2 presents the staggered SDID estimates of the association between QSW staffing and nursing home outcomes between 2017 and 2021. The estimates for successful discharge rates (−0.2 percentage points; 95% CI, −1.0 to 0.6 percentage points) and hospitalizations per resident-year (1.0 percentage points; 95% CI, −1.3 to 3.4 percentage points) were small and not statistically significant; for 30-day rehospitalization, the point estimate suggested a 0.4–percentage point decrease (95% CI, −0.8 to 0.1 percentage points), about 2% of the 2017 mean (*P* = .12). The primary outcome, restraint use, showed a more substantive association. Restraint use was 0.3 percentage points lower (95% CI, −0.6 to −0.0 percentage points), or 46% of the 2017 mean. Restraint use was rare during the study period—below 1% at baseline and decreasing over time—and the estimated association should be interpreted in this context. These findings suggested that QSW staffing was associated with reduced restraint use, and point estimates suggested lower rehospitalization rates, with little association for hospitalizations or discharge outcomes. We present an event-study plot for restraint use in Figure 2, which shows no evidence of differential pretreatment trends and a downward posttreatment pattern, with estimated coefficients statistically significant in the first and third year after QSW staffing. Event-study plots for the 3 secondary outcomes are shown in eFigures 1, 2, and 3 in Supplement 1. Joint *F* tests of the pretreatment coefficients were not statistically significant at the 5% level for all 4 outcomes, supporting the parallel trends assumption.

**Table 2.** Staggered SDID Estimates of Qualified Social Worker Employment and Nursing Home Outcomes, 2017-2021

Outcome variable^a^	Coefficient^b^	Bootstrap SE^c^	*P* value	Mean^d^
**Pooled sample (2491 facilities)**				
Restraint	−0.003	0.002	.04	0.007
Hospitalization	0.010	0.012	.40	1.123
Rehospitalization	−0.004	0.002	.12	0.179
Discharge	−0.002	0.004	.62	0.546
**Facilities with proportion of residents with ADRD above the median** ^e^				
Restraint	−0.005	0.002	.02	0.009
Hospitalization	0.002	0.015	.88	0.963
Rehospitalization	−0.004	0.004	.29	0.183
Discharge	−0.007	0.006	.31	0.483
**Facilities with proportion of residents with ADRD below the median** ^f^				
Restraint	−0.001	0.002	.55	0.005
Hospitalization	0.015	0.018	.42	1.292
Rehospitalization	−0.004	0.003	.16	0.174
Discharge	0.003	0.005	.55	0.613

Abbreviations: ADRD, Alzheimer disease and related dementias; SDID, synthetic difference-in-differences.

^a^ We apply the SDID method with staggered adoption to the facility-level balanced panel data. The analytic sample includes 2491 facilities observed over 5 years (2017-2021). The SDID specification incorporates facility fixed effects and year fixed effects and uses data-driven unit and time weights to align treated and control facilities on pretreatment outcome trajectories. Restraint is the proportion of residents restrained at the annual certification survey. Hospitalization is the number of hospital transfers from the facility during the year divided by total resident-years. Rehospitalization is the proportion of new postacute admissions from hospitals transferred to an acute hospital within 30 days. Discharge is the proportion of new postacute admissions from hospitals discharged alive to the community within 100 days, without a subsequent nursing home admission within 30 days.

^b^ The estimated coefficients for the association between employing a qualified social worker and each of the 4 outcome variables.

^c^ Standard errors are calculated using the bootstrap procedure, with the corresponding *P* values reported.

^d^ The mean of each outcome variable in 2017.

^e^ Restricts the sample to facilities with above-median prevalence of ADRD.

^f^ Restricts the sample to facilities with below-median prevalence of ADRD.

We next examined heterogeneity by 2017 ADRD prevalence, dividing facilities into high-prevalence (above median) and low-prevalence (at or below median) groups (Table 2). Restraint use was lower by 0.5 percentage points (95% CI, −0.9 to −0.1 percentage points)—58% of the 2017 average—among high-prevalence facilities, with no detectable association in low-prevalence facilities. No statistically significant associations were found for hospitalization, rehospitalization, or discharge outcomes in either subgroup. These findings suggested that the benefits associated with QSW staffing were concentrated in settings with greater dementia care needs, where psychosocial support was critical for reducing use of restraints.

### Sensitivity Analysis

As a sensitivity analysis, we increased the QSW employment threshold to 1 hour per day. Under this definition, a facility was classified as treated if its annual mean daily employee QSW hours exceed 1. This reduced the sample to 2250 facilities, of which 836 employed a QSW between 2018 and 2021. As shown in Table 3, the estimated associations for hospitalization and discharge outcomes remained near 0 and statistically insignificant, consistent with the main results in Table 2. For the 30-day rehospitalization rate, the estimate is −0.4 percentage points (95% CI, −0.9 to 0.1 percentage points; *P* = .10), which provides suggestive evidence of a negative association (Table 3). For the restraint use, the estimate is −0.2 percentage points (95% CI, −0.5 to 0.0 percentage points), smaller than the main estimate and statistically insignificant at the 5% level.

**Table 3.** Sensitivity Analysis: Staggered SDID Estimates of Qualified Social Worker Employment and Nursing Home Outcomes, 2017-2021

Outcome variable^a^	Coefficient^b^	Bootstrap SE^c^	*P* value	Mean^d^
**Pooled sample (2250 facilities)**				
Restraint	−0.002	0.001	.09	0.007
Hospitalization	0.005	0.014	.74	1.118
Rehospitalization	−0.004	0.002	.10	0.178
Discharge	−0.002	0.004	.68	0.547
**Facilities with proportion of residents with ADRD above the median** ^e^				
Restraint	−0.006	0.003	.03	0.009
Hospitalization	0.005	0.064	.77	0.960
Rehospitalization	−0.004	0.004	.36	0.183
Discharge	−0.008	0.075	.27	0.482
**Facilities with proportion of residents with ADRD below the median** ^f^				
Restraint	0.001	0.001	.69	0.004
Hospitalization	0.002	0.021	.94	1.285
Rehospitalization	−0.005	0.003	.07	0.173
Discharge	0.005	0.005	.38	0.616

Abbreviations: ADRD, Alzheimer disease and related dementias; SDID, synthetic difference-in-differences.

^a^ We used the SDID approach with staggered adoption to the facility-level balanced panel data. The sample includes 2250 facilities observed over 5 years (2017-2021). The treatment variable equals 1 if a facility’s annual mean of total daily hours worked by employed (noncontract) qualified social workers was greater than 1, and 0 if the annual mean was 0. The SDID specification incorporates facility fixed effects and year fixed effects and uses data-driven unit and time weights to align treated and control facilities on pretreatment outcome trajectories. Restraint is the proportion of residents restrained at the annual certification survey. Hospitalization is the number of hospital transfers from the facility during the year divided by total resident-years. Rehospitalization is the proportion of new postacute admissions from hospitals transferred to an acute hospital within 30 days. Discharge is the proportion of new postacute admissions from hospitals discharged alive to the community within 100 days, without a subsequent nursing home admission within 30 days.

^b^ The estimated coefficients for the association between employing a qualified social worker and each of the 4 outcome variables.

^c^ Standard errors are calculated using the bootstrap procedure, with the corresponding *P* values reported.

^d^ The mean of each outcome variable in 2017.

^e^ Restricts the sample to facilities with above-median prevalence of ADRD.

^f^ Restricts to those with below-median prevalence of ADRD.

Table 3 also shows the subgroup results. The decrease in rehospitalization rate was concentrated among low-prevalence facilities (−0.5 percentage points; 95% CI, −1.1 to 0.0 percentage points; *P* = .07), while the reduction in restraint use continued to be observed primarily in high-prevalence facilities (−0.6 percentage points; 95% CI, −1.1 to −0.0 percentage points; *P* = .03). One possible explanation is that QSWs may support care coordination and discharge planning among residents without severe cognitive impairment, whereas their role in dementia care is reflected more strongly in reduced reliance on restraints.^19^ Taken together, these findings indicated that the main results were insensitive to alternative definitions of QSW employment.

### Robustness Checks

We conducted 2 robustness checks to assess the stability of the main findings under alternative specifications. First, we reestimated the SDID models including additional staffing controls (mean daily registered nurse, licensed practical nurse, and certified nursing assistant hours, as well as contract QSW hours) and measures of local COVID-19 intensity (ie, annual mean daily state-level COVID-19 confirmed cases and deaths). As reported in eTable 3 in Supplement 1, the estimates were qualitatively and quantitatively similar to the main findings, suggesting that the results were not associated with differences in other staffing levels or pandemic-related local shocks. Second, we used the imputation-based difference-in-differences estimator,^26^ which imputes counterfactual outcomes using unit and time fixed effects estimated from untreated observations. The resulting estimates were consistent with the primary SDID results (eTable 4 in Supplement 1). We also present imputation-based event-study plots that show no evidence of differential pretreatment trends (eFigures 4-7 in Supplement 1), further supporting the parallel trends assumption.

## Discussion

In this national cohort study of US nursing homes, we found that employment of QSWs was associated with reduced restraint use, and the point estimate suggested lower 30-day rehospitalization rates; no significant associations were observed for overall hospitalizations or successful discharges. The association with restraint use was especially pronounced in facilities with a higher prevalence of ADRD, suggesting an important role for QSWs in settings with greater resident psychosocial and behavioral care needs. These findings extended prior descriptive research by providing, to our knowledge, the first national quasi-experimental evidence on QSW staffing in nursing homes. The observed associations may reflect QSWs’ roles in psychosocial assessment, individualized care planning, and advocacy for less-restrictive care,^27^ as well as potential contributions to discharge coordination and family communication, although the estimated association with rehospitalization was not statistically significant.

The results have several policy implications. First, although federal regulations require facilities with more than 120 beds to employ a QSW, compliance remains incomplete, and smaller facilities are exempt from this requirement. Strengthening enforcement and considering expansion of these standards could improve access to social work services.^28^ Second, the observed associations with reduced restraint use and modest decreases in rehospitalizations align with Medicare and Medicaid quality-based payment initiatives, suggesting that expanding social work capacity may support both resident well-being and cost-containment goals. Third, stronger associations in facilities with a high prevalence of ADRD underscore the importance of dementia-focused staffing policies, where psychosocial support is particularly critical to quality of care.

### Limitations

This study has some limitations. First, the analytic sample excluded facilities that had already employed QSWs in 2017, as well as facilities that entered and exited QSW employment during the study period. As a result, the findings reflect associations among nursing homes that newly adopted and retained social work staffing, a nonrandom subset of US facilities, and should not be extrapolated to facilities with longstanding or intermittent social work capacity. Although these restrictions were necessary to construct a valid counterfactual under the staggered-adoption SDID framework, they limit external validity and may underestimate broader associations. Future research using alternative quasi-experimental designs, such as policy-driven natural experiments or instrumental variables, could incorporate facilities with preexisting or nonmonotonic QSW staffing. Second, although the SDID approach strengthens the parallel trends assumption, it cannot fully account for unobserved time-varying factors, such as local labor market shocks (eg, QSW availability) or state-level policy changes, that may simultaneously influence both QSW employment and resident outcomes. Third, physical restraint use was relatively rare during the study period; although it is a clinically and regulatorily salient indicator of person-centered, less-restrictive care, its low prevalence may limit statistical power and suggests that estimates should be interpreted as changes in a narrowly defined quality indicator rather than overall nursing home quality. Fourth, our outcomes capture clinical and regulatory indicators but do not measure psychosocial well-being, family engagement, or staff satisfaction, which future research could incorporate.

## Conclusions

In this cohort study of US nursing homes, QSW staffing was associated with reduced restraint use, particularly in facilities with a higher prevalence of ADRD. These findings highlight the potential role of social work staffing in supporting person-centered, less-restrictive care. Future research should examine broader effects on psychosocial outcomes, staff well-being, and long-term facility performance.

## Notes

Supplement 1. **eFigure 1.** Event-Study Estimates of the Association Between Qualified Social Worker Employment and Hospitalization, 2017-2021 (SDID) **eFigure 2.** Event-Study Estimates of the Association Between Qualified Social Worker Employment and Rehospitalization, 2017-2021 (SDID) **eFigure 3.** Event-Study Estimates of the Association Between Qualified Social Worker Employment and Discharge, 2017-2021 (SDID) **eFigure 4.** Event-Study Estimates of the Association Between Qualified Social Worker Employment and Resident Restraint Use, 2017-2021 (Imputation-Based Method) **eFigure 5.** Event-Study Estimates of the Association Between Qualified Social Worker Employment and Hospitalization, 2017-2021 (Imputation-Based Method) **eFigure 6.** Event-Study Estimates of the Association Between Qualified Social Worker Employment and Rehospitalization, 2017-2021 (Imputation-Based Method) **eFigure 7.** Event-Study Estimates of the Association Between Qualified Social Worker Employment and Discharge, 2017-2021 (Imputation-Based Method) **eTable 1.** Summary Statistics of Facility Characteristics: Facilities Included vs Excluded (In-Out), 2017 **eTable 2.** Summary Statistics of Facility Characteristics: Control vs Treated Facilities in 2017 **eTable 3.** SDID Estimates of Qualified Social Worker Employment and Nursing Home Outcomes, With and Without Additional Controls, 2017-2021 **eTable 4.** SDID and BJS Estimates of Qualified Social Worker Employment and Nursing Home Outcomes, 2017-2021 **eMethods.** Synthetic Difference-in-Differences with Staggered Adoption
eReferences.

Supplement 2.
Data Sharing Statement
