# Supplementary material for: Staffing of Qualified Social Workers and Nursing Home Quality of Care
Source: JAMA Netw Open. 2026 Feb 25;9(2):e260074. doi: 10.1001/jamanetworkopen.2026.0074 (PMC12936878; doi:10.1001/jamanetworkopen.2026.0074)
Supplement: Supplement 1. — eFigure 1. Event-Study Estimates of the Association Between Qualified Social Worker Employment and Hospitalization, 2017-2021 (SDID) eFigure 2. Event-Study Estimates of the Association Between Qualified Social Worker Employment and Rehospitalization, 2017-2021 (SDID) eFigure 3. Event-Study Estimates of the Association Between Qualified Social Worker Employment and Discharge, 2017-2021 (SDID) eFigure 4. Event-Study Estimates of the Association Between Qualified Social Worker Employment and Resident Restraint Use, 2017-2021 (Imputation-Based Method) eFigure 5. Event-Study Estimates of the Association Between Qualified Social Worker Employment and Hospitalization, 2017-2021 (Imputation-Based Method) eFigure 6. Event-Study Estimates of the Association Between Qualified Social Worker Employment and Rehospitalization, 2017-2021 (Imputation-Based Method) eFigure 7. Event-Study Estimates of the Association Between Qualified Social Worker Employment and Discharge, 2017-2021 (Imputation-Based Method) eTable 1. Summary Statistics of Facility Characteristics: Facilities Included vs Excluded (In-Out), 2017 eTable 2. Summary Statistics of Facility Characteristics: Control vs Treated Facilities in 2017 eTable 3. SDID Estimates of Qualified Social Worker Employment and Nursing Home Outcomes, With and Without Additional Controls, 2017-2021 eTable 4. SDID and BJS Estimates of Qualified Social Worker Employment and Nursing Home Outcomes, 2017-2021 eMethods. Synthetic Difference-in-Differences with Staggered Adoption eReferences. [file jamanetwopen-e260074-s001.pdf]

Supplementary Online Content

Chen Y, Xu L, Jiang W, Fields N. Staffing of qualified social workers and nursing home quality of care. *JAMA Netw Open.* 2026;9(2):e260074. doi:10.1001/jamanetworkopen.2026.0074

eFigure 1. Event-Study Estimates of the Association Between Qualified Social Worker Employment and Hospitalization, 2017-2021 (SDID)

eFigure 2. Event-Study Estimates of the Association Between Qualified Social Worker Employment and Rehospitalization, 2017-2021 (SDID)

eFigure 3. Event-Study Estimates of the Association Between Qualified Social Worker Employment and Discharge, 2017-2021 (SDID)

eFigure 4. Event-Study Estimates of the Association Between Qualified Social Worker Employment and Resident Restraint Use, 2017-2021 (Imputation-Based Method)

eFigure 5. Event-Study Estimates of the Association Between Qualified Social Worker Employment and Hospitalization, 2017-2021 (Imputation-Based Method)

eFigure 6. Event-Study Estimates of the Association Between Qualified Social Worker Employment and Rehospitalization, 2017-2021 (Imputation-Based Method)

eFigure 7. Event-Study Estimates of the Association Between Qualified Social Worker Employment and Discharge, 2017-2021 (Imputation-Based Method)

eTable 1. Summary Statistics of Facility Characteristics: Facilities Included vs Excluded (In-Out), 2017

eTable 2. Summary Statistics of Facility Characteristics: Control vs Treated Facilities in 2017

eTable 3. SDID Estimates of Qualified Social Worker Employment and Nursing Home Outcomes, With and Without Additional Controls, 2017-2021

eTable 4. SDID and BJS Estimates of Qualified Social Worker Employment and Nursing Home Outcomes, 2017-2021

eMethods. Synthetic Difference-in-Differences with Staggered Adoption

eReferences.

This supplementary material has been provided by the authors to give readers additional information about their work.

© 2026 Chen Y et al. *JAMA Network Open.*

eFigure 1. Event-Study Estimates of the Association Between Qualified Social Worker Employment and Hospitalization, 2017-2021 (SDID)

Notes: The figure reports event study estimates using the synthetic difference-in-differences method with staggered adoption (Arkhangelsky et al., 2021; Clarke et al., 2024). The analytic sample consists of 2,491 nursing facilities observed annually from 2017 through 2021. The outcome variable is the number of hospital transfers from the facility during the year divided by total resident-years. The horizontal red dashed line represents zero. The vertical black line at Years to Treatment = 0 corresponds to the year in which a facility first employs a qualified social worker. The period Years to Treatment = -1 serves as the reference (baseline) year. Shaded gray areas indicate 95% confidence intervals. We conduct a joint F test of the pre-treatment coefficients, which failed to reject the null hypothesis of no differential pre-treatment trends at the 5% level (*P*=0.70).

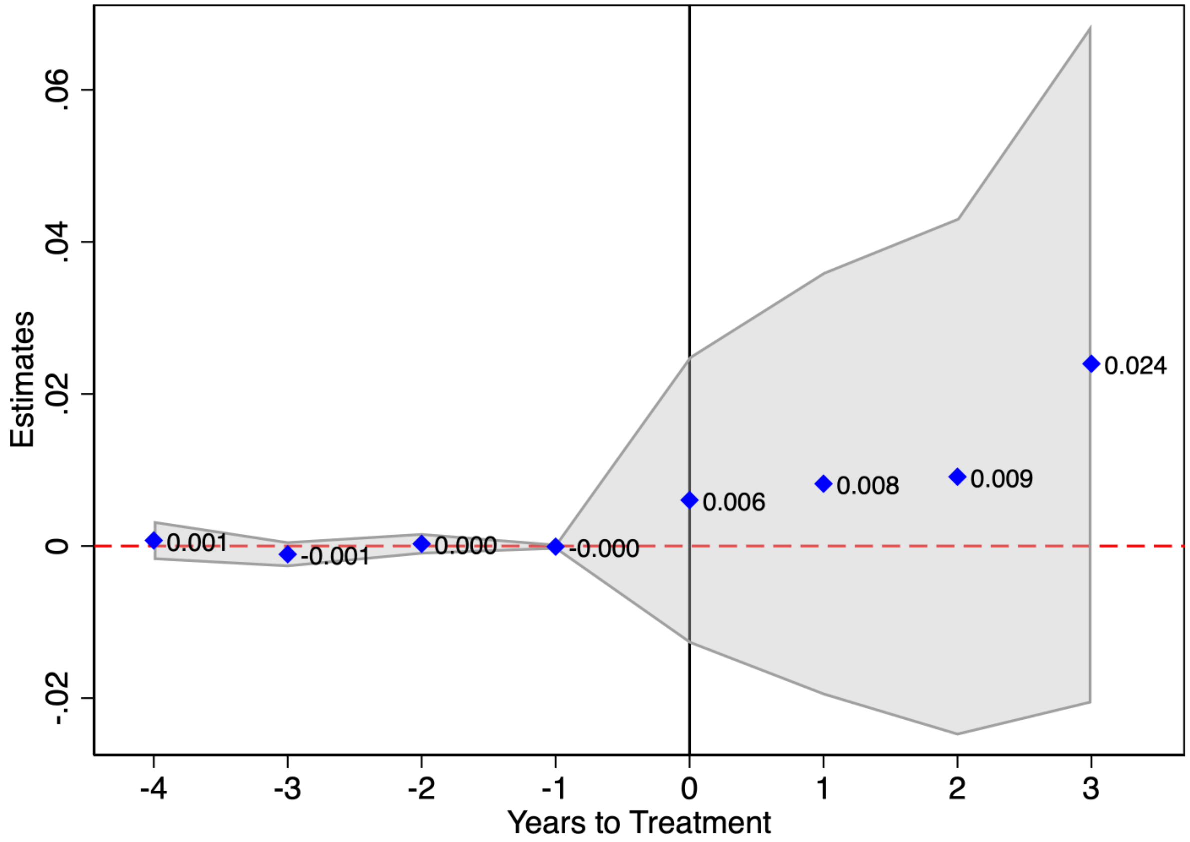

© 2026 Chen Y et al. *JAMA Network Open.*

eFigure 2. Event-Study Estimates of the Association Between Qualified Social Worker Employment and Rehospitalization, 2017-2021 (SDID)

<u>Notes</u>: The figure reports event study estimates using the synthetic difference-in-differences method with staggered adoption (Arkhangelsky et al., 2021; Clarke et al., 2024). The analytic sample consists of 2,491 nursing facilities observed annually from 2017 through 2021. The outcome variable is the proportion of new post-acute admissions from hospitals transferred to an acute hospital within 30 days. The horizontal red dashed line represents zero. The vertical black line at Years to Treatment = 0 corresponds to the year in which a facility first employs a qualified social worker. The period Years to Treatment = -1 serves as the reference (baseline) year. Shaded gray areas indicate 95% confidence intervals. We conduct a joint F test of the pre-treatment coefficients, which failed to reject the null hypothesis of no differential pre-treatment trends at the 5% level (*P*=0.09).

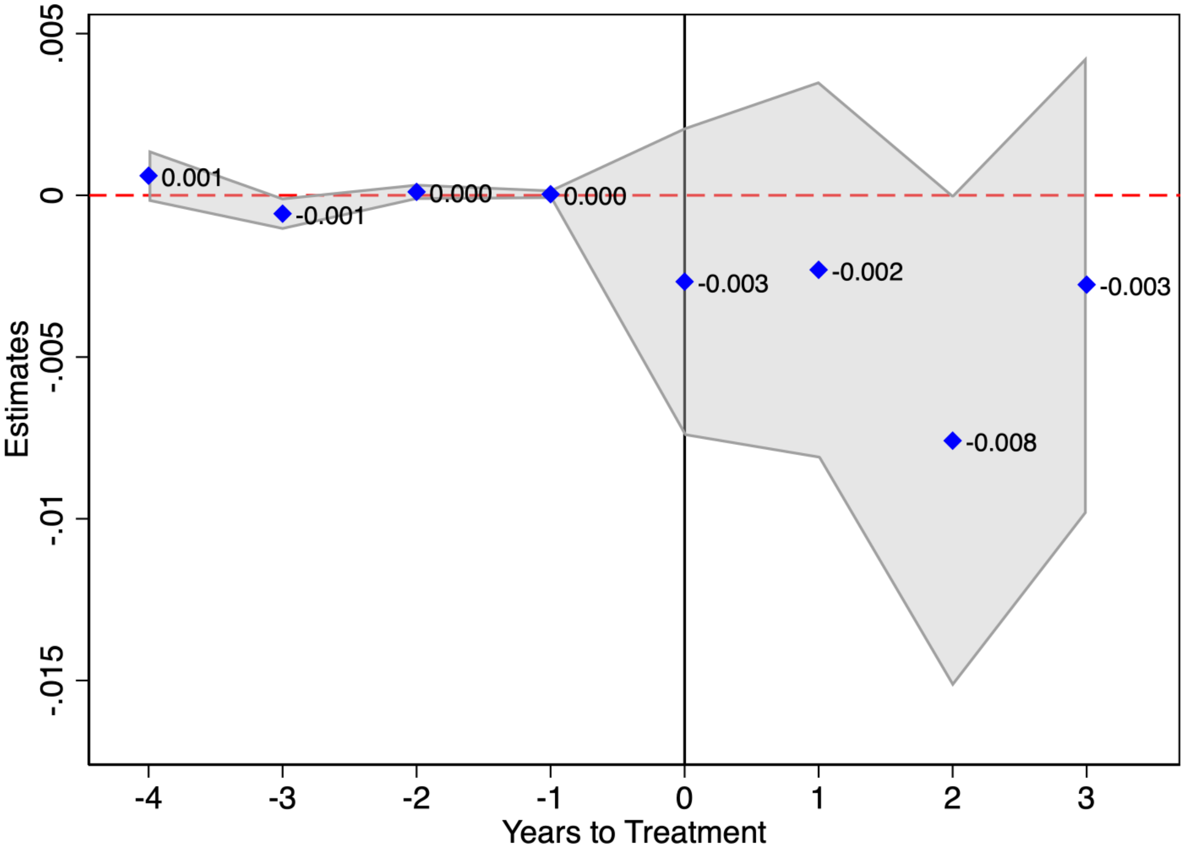

© 2026 Chen Y et al. *JAMA Network Open.*

eFigure 3. Event-Study Estimates of the Association Between Qualified Social Worker Employment and Discharge, 2017-2021 (SDID)

<u>*Notes*</u>: The figure reports event study estimates using the synthetic difference-in-differences method with staggered adoption (Arkhangelsky et al., 2021; Clarke et al., 2024). The analytic sample consists of 2,491 nursing facilities observed annually from 2017 through 2021. The outcome variable is the proportion of new post-acute admissions from hospitals discharged alive to the community within 100 days, without a subsequent nursing home admission within 30 days. The horizontal red dashed line represents zero. The vertical black line at Years to Treatment = 0 corresponds to the year in which a facility first employs a qualified social worker. The period Years to Treatment = -1 serves as the reference (baseline) year. Shaded gray areas indicate 95% confidence intervals. We conduct a joint F test of the pre-treatment coefficients, which failed to reject the null hypothesis of no differential pre-treatment trends at the 5% level (*P*=0.20).

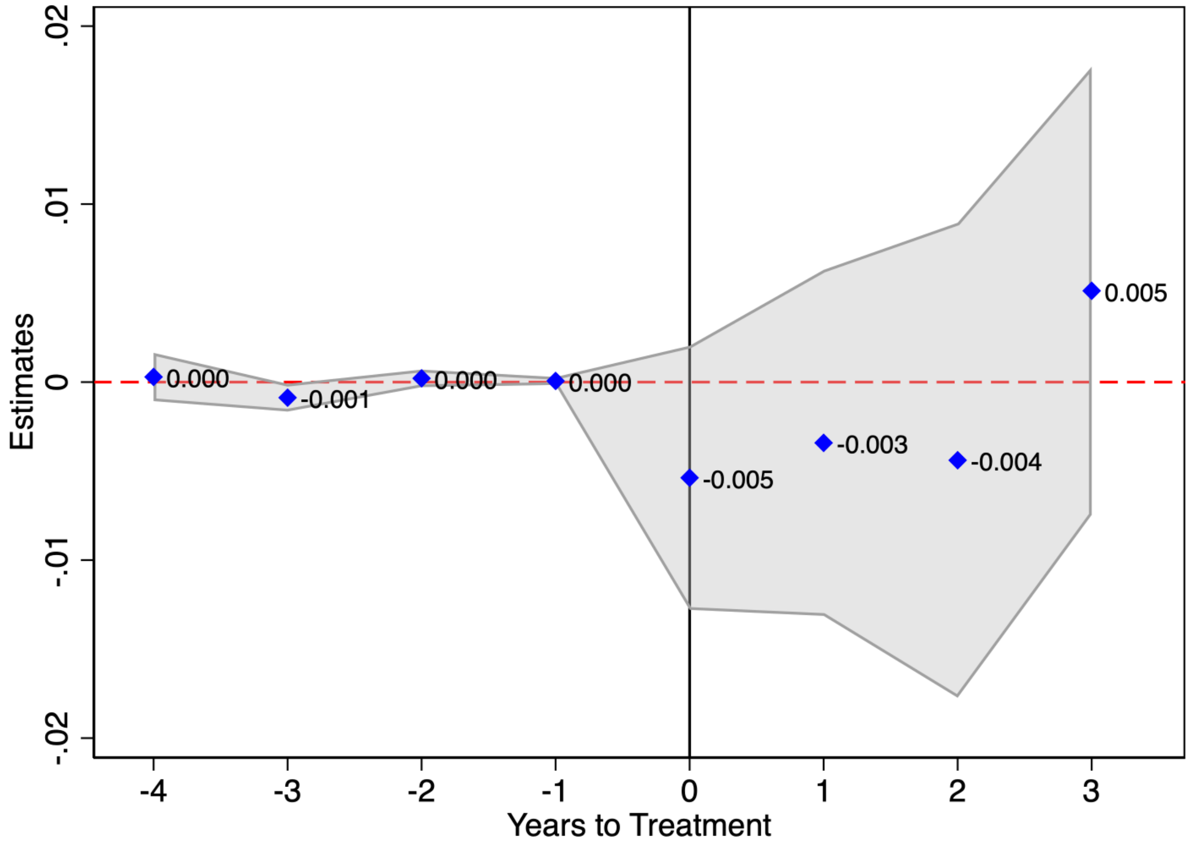

© 2026 Chen Y et al. *JAMA Network Open.*

eFigure 4. Event-Study Estimates of the Association Between Qualified Social Worker Employment and Resident Restraint Use, 2017-2021 (Imputation-Based Method)

Notes: The figure reports event study estimates using the imputation estimator. The analytic sample consists of 2,491 nursing facilities observed annually from 2017 through 2021. The outcome variable is the proportion of residents restrained at the annual certification survey. The horizontal gray dashed line represents zero. Blue markers denote pre-treatment periods (-3 to -1) and pink markers denote post-treatment periods (0 to 3). The point at Years to Treatment = 0 corresponds to the year in which the facility starts the employment of a qualified social worker. Confidence intervals are at the 95 percent. We conduct a joint F test of the pre-treatment coefficients, which failed to reject the null hypothesis of no differential pre-treatment trends at the 5% level.

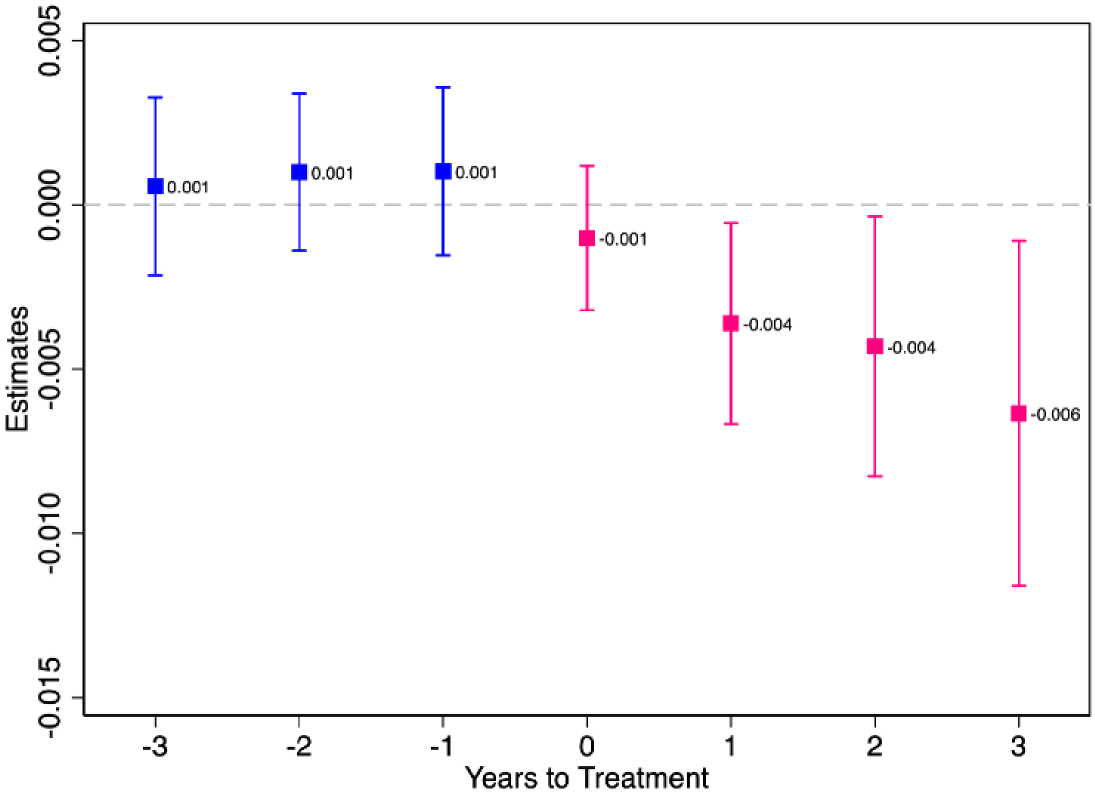

eFigure 5. Event-Study Estimates of the Association Between Qualified Social Worker Employment and Hospitalization, 2017-2021 (Imputation-Based Method)

Notes: The figure reports event study estimates using the imputation estimator. The analytic sample consists of 2,491 nursing facilities observed annually from 2017 through 2021. The outcome variable is the number of hospital transfers from the facility during the year divided by total resident-years. The horizontal red dashed line represents zero. The horizontal gray dashed line represents zero. Blue markers denote pre-treatment periods (-3 to -1) and pink markers denote post-treatment periods (0 to 3). The point at Years to Treatment = 0 corresponds to the year in which the facility starts the employment of a qualified social worker. Confidence intervals are at the 95 percent. We conduct a joint F test of the pre-treatment coefficients, which failed to reject the null hypothesis of no differential pre-treatment trends at the 5% level.

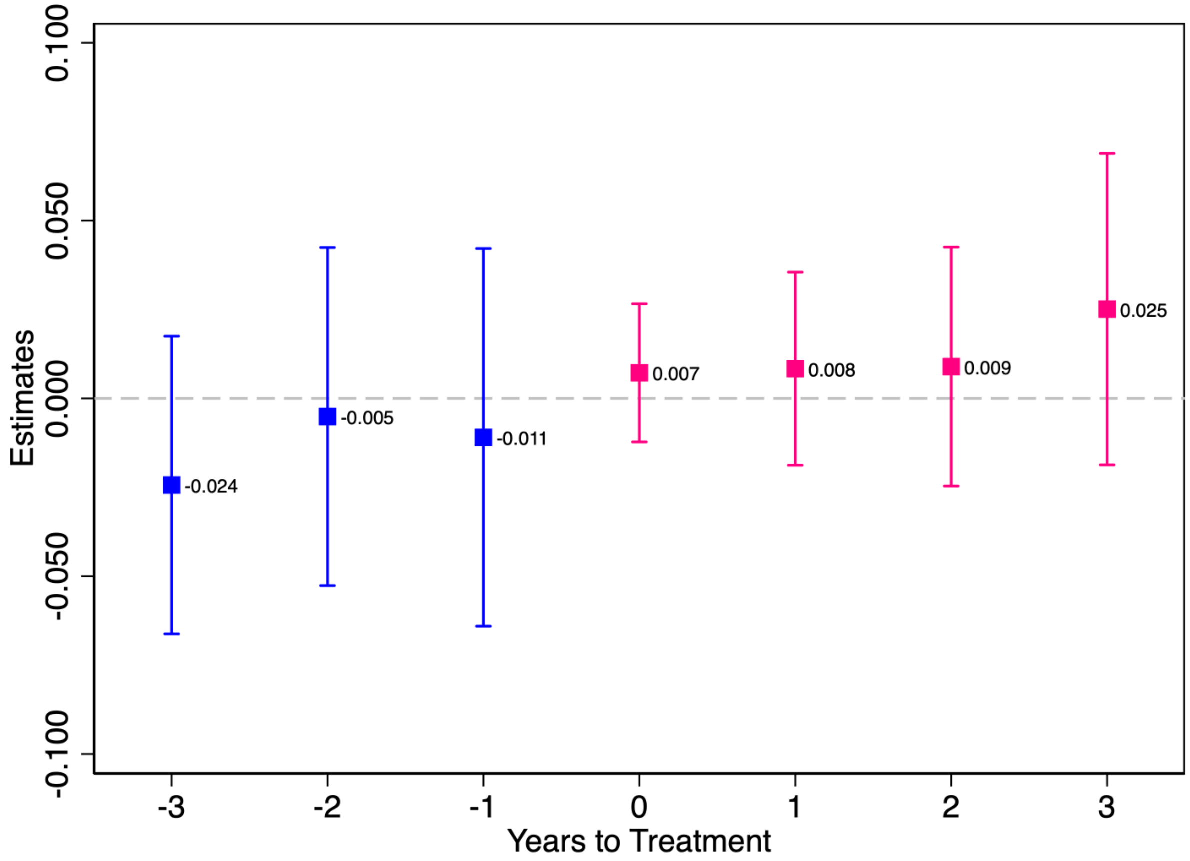

© 2026 Chen Y et al. *JAMA Network Open.*

eFigure 6. Event-Study Estimates of the Association Between Qualified Social Worker Employment and Rehospitalization, 2017-2021 (Imputation-Based Method)

Notes: The figure reports event study estimates using the imputation estimator. The analytic sample consists of 2,491 nursing facilities observed annually from 2017 through 2021. The outcome variable is the proportion of new post-acute admissions from hospitals transferred to an acute hospital within 30 days. The horizontal gray dashed line represents zero. Blue markers denote pre-treatment periods (-3 to -1) and pink markers denote post-treatment periods (0 to 3). The point at Years to Treatment = 0 corresponds to the year in which the facility starts the employment of a qualified social worker. Confidence intervals are at the 95 percent. We conduct a joint F test of the pre-treatment coefficients, which failed to reject the null hypothesis of no differential pre-treatment trends at the 5% level.

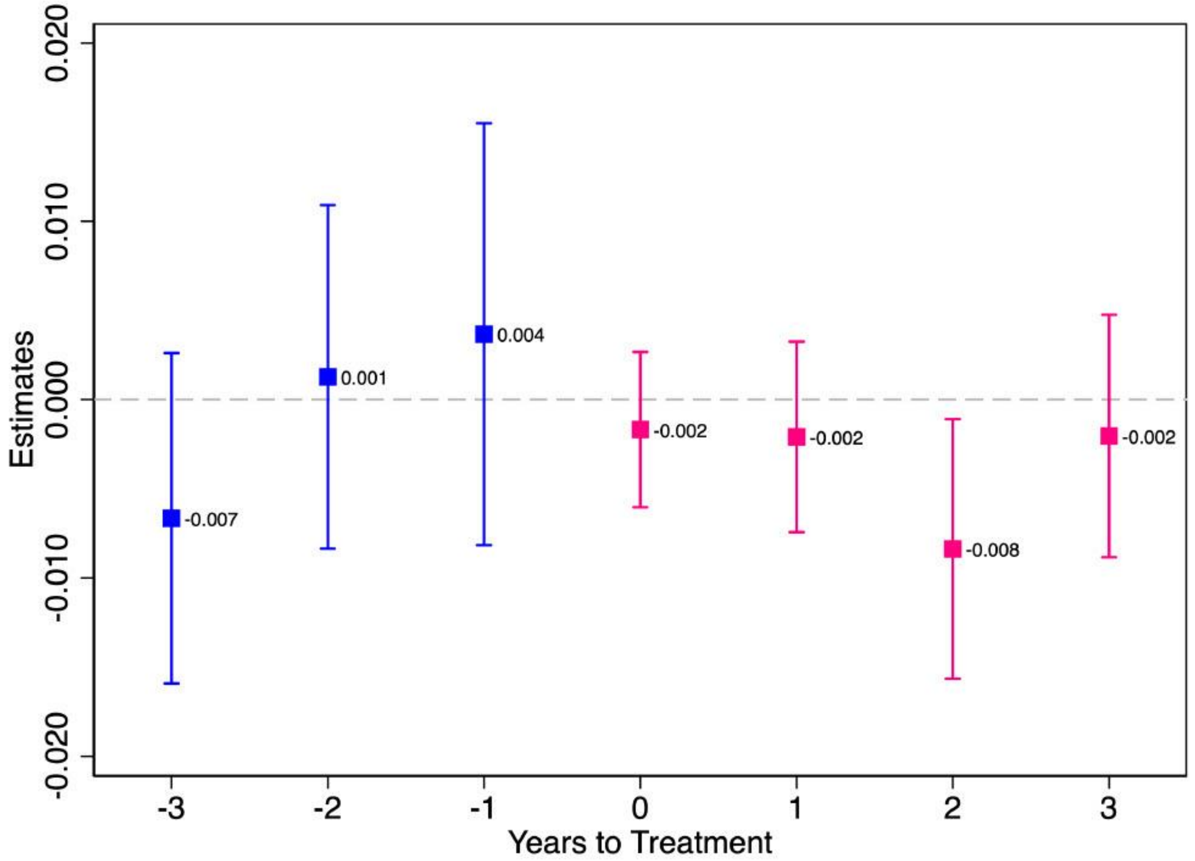

© 2026 Chen Y et al. *JAMA Network Open.*

eFigure 7. Event-Study Estimates of the Association Between Qualified Social Worker Employment and Discharge, 2017-2021 (Imputation-Based Method)

Notes: The figure reports event study estimates using the imputation estimator. The analytic sample consists of 2,491 nursing facilities observed annually from 2017 through 2021. The outcome variable is the proportion of new post-acute admissions from hospitals discharged alive to the community within 100 days, without a subsequent nursing home admission within 30 days. The horizontal red dashed line represents zero. The horizontal gray dashed line represents zero. Blue markers denote pre-treatment periods (-3 to -1) and pink markers denote post-treatment periods (0 to 3). The point at Years to Treatment = 0 corresponds to the year in which the facility starts the employment of a qualified social worker. Confidence intervals are at the 95 percent. We conduct a joint F test of the pre-treatment coefficients, which failed to reject the null hypothesis of no differential pre-treatment trends at the 5% level.

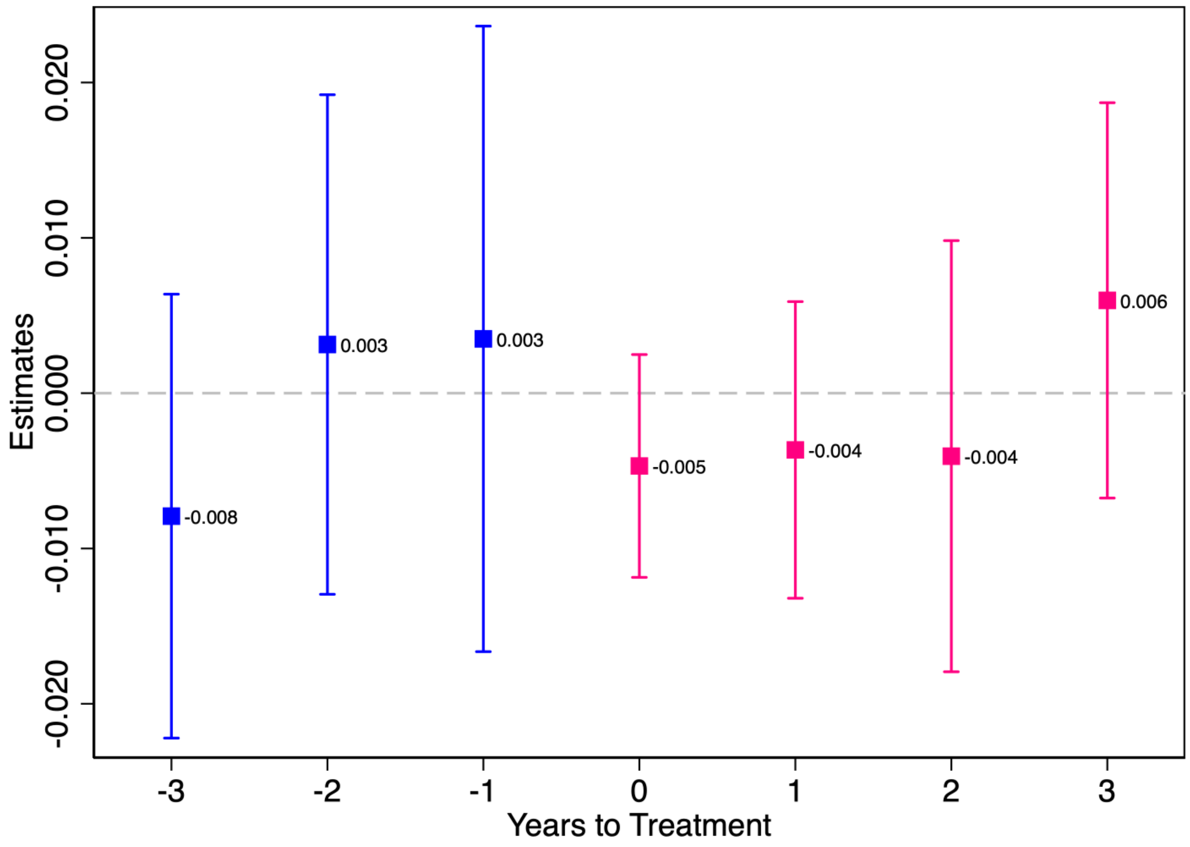

© 2026 Chen Y et al. *JAMA Network Open.*

eTable 1. Summary Statistics of Facility Characteristics: Facilities Included vs Excluded (In-Out), 2017

	(1)		(2)		(3)	(4)
	Included		Excluded (In-Out)		Difference	
Variable	N	Mean/SD	N	Mean/SD	(1)-(2)	*P* value
Total number of beds	2491	100.418<br>(43.466)	266	98.308<br>(40.667)	2.109<br>(2.787)	0.45
Has Alzheimer's disease SCU	2491	0.137<br>(0.344)	266	0.128<br>(0.335)	0.009<br>(0.022)	0.67
Has any SCU	2491	0.149<br>(0.356)	266	0.143<br>(0.351)	0.006<br>(0.023)	0.80
Chain affiliation	2491	0.625<br>(0.484)	266	0.605<br>(0.490)	0.019<br>(0.031)	0.54
For-profit	2491	0.797<br>(0.402)	266	0.801<br>(0.400)	-0.004<br>(0.026)	0.88
Hospital-based	2491	0.015<br>(0.123)	266	0.019<br>(0.136)	-0.004<br>(0.008)	0.66
Average age of residents	2491	78.974<br>(5.922)	266	78.735<br>(5.768)	0.239<br>(0.381)	0.53
Medicaid share of residents	2491	0.613<br>(0.212)	266	0.626<br>(0.201)	-0.013<br>(0.014)	0.34
Medicare share of residents	2491	0.135<br>(0.112)	266	0.129<br>(0.094)	0.005<br>(0.007)	0.46
RN hours per resident-day	2491	0.357<br>(0.267)	266	0.339<br>(0.253)	0.018<br>(0.017)	0.29
LPN hours per resident-day	2491	0.804<br>(0.319)	266	0.838<br>(0.361)	-0.034<br>(0.021)	0.10
CNA hours per resident-day	2491	2.187<br>(0.514)	266	2.151<br>(0.534)	0.037<br>(0.033)	0.27

Notes: We compare the 2,491 included facilities with facilities that had complete five-year data, did not employ a QSW in 2017, but entered and exited QSW employment between 2017 and 2021. The variables reported include the total number of beds; the presence of an Alzheimer's disease Special Care Unit (SCU); the presence of any SCU; chain affiliation; for-profit status; hospital-based status; the average age of residents; the share of residents whose primary support is Medicaid; the share whose primary support is Medicare; and the average weekday hours per resident provided by registered nurses (RN), licensed practical nurses (LPN), and certified nursing assistants (CNA). Column 3 reports the mean differences between facilities excluded from the main analysis and those included, along with corresponding standard errors from a two-sample t-test assuming equal variances. Column 4 report the associated *P* value.

© 2026 Chen Y et al. *JAMA Network Open.*

eTable 2. Summary Statistics of Facility Characteristics: Control vs Treated Facilities in 2017

	(1)		(2)		(3)	(4)
	Control		Treated		Difference	
Variable	N	Mean/SD	N	Mean/SD	(1)-(2)	*P* value
Total number of beds	1414	94.897	1077	107.666	-12.769	<0.001
		(41.070)		(45.437)	(1.740)	
Has Alzheimer's disease SCU	1414	0.138	1077	0.136	0.001	0.92
		(0.345)		(0.343)	(0.014)	
Has any SCU	1414	0.152	1077	0.144	0.008	0.57
		(0.359)		(0.351)	(0.014)	
Chain affiliation	1414	0.585	1077	0.677	-0.092	<0.001
		(0.493)		(0.468)	(0.020)	
For-profit	1414	0.776	1077	0.825	-0.049	0.002
		(0.417)		(0.381)	(0.016)	
Hospital-based	1414	0.018	1077	0.011	0.007	0.14
		(0.134)		(0.105)	(0.005)	
Average age of residents	1414	79.431	1077	78.374	1.057	<0.001
		(6.009)		(5.754)	(0.239)	
Medicaid share of residents	1414	0.609	1077	0.620	-0.011	0.20
		(0.216)		(0.207)	(0.009)	
Medicare share of residents	1414	0.130	1077	0.140	-0.010	0.03
		(0.113)		(0.111)	(0.005)	
RN hours per resident-day	1414	0.351	1077	0.365	-0.013	0.22
		(0.277)		(0.254)	(0.011)	
LPN hours per resident-day	1414	0.792	1077	0.820	-0.029	0.03
		(0.320)		(0.317)	(0.013)	
CNA hours per resident-day	1414	2.180	1077	2.197	-0.017	0.42
		(0.527)		(0.496)	(0.021)	

Notes: The variables reported include the total number of beds; the presence of an Alzheimer's disease Special Care Unit (SCU); the presence of any SCU; chain affiliation; for-profit status; hospital-based status; the average age of residents; the share of residents whose primary support is Medicaid; the share whose primary support is Medicare; and the average weekday hours per resident provided by registered nurses (RN), licensed practical nurses (LPN), and certified nursing assistants (CNA). Column 3 reports the mean differences between facilities excluded from the main analysis and those included, along with corresponding standard errors from a two-sample t-test assuming equal variances. Column 4 report the associated *P* value.

© 2026 Chen Y et al. *JAMA Network Open.*

eTable 3. SDID Estimates of Qualified Social Worker Employment and Nursing Home Outcomes, With and Without Additional Controls, 2017-2021

	(1)	(2)	(3)	(4)	(5)	(6)
	Main Specification			With Additional Controls		
	Estimate	SE	*P* value	Estimate	SE	*P* value
Restraint	-0.003	(0.002)	0.04	-0.003	(0.002)	0.04
Hospitalization	0.010	(0.012)	0.40	0.009	(0.012)	0.48
Rehospitalization	-0.004	(0.002)	0.12	-0.003	(0.002)	0.13
Discharge	-0.002	(0.004)	0.62	-0.002	(0.004)	0.61

Notes: We apply the synthetic difference-in-differences (SDID) estimator to a balanced facility-level panel. The analytic sample consists of 2,491 nursing facilities observed annually from 2017 through 2021. Columns 1-3 report SDID estimates of the association between employing a qualified social worker and each outcome without additional covariates, while Columns 4-6 report estimates from specifications that include covariates. These covariates include average weekday hours per resident provided by registered nurses, licensed practical nurses, and certified nursing assistants; average daily contract hours of qualified social workers; and annual average daily state-level COVID-19 confirmed cases and deaths from Google's COVID-19 Open Data. "Restraint" = The proportion of residents restrained at the annual certification survey. "Hospitalization" = The number of hospital transfers from the facility during the year divided by total resident-years. "Rehospitalization" = The proportion of new post-acute admissions from hospitals transferred to an acute hospital within 30 days. "Discharge" = The proportion of new post-acute admissions from hospitals discharged alive to the community within 100 days, without a subsequent nursing home admission within 30 days. Standard errors are obtained using bootstrap inference for SDID and cluster-robust inference at the facility level for the imputation estimates.

© 2026 Chen Y et al. *JAMA Network Open.*

eTable 4. SDID and BJS Estimates of Qualified Social Worker Employment and Nursing Home Outcomes, 2017-2021

	(1)	(2)	(3)	(4)	(5)	(6)
		SDID			BJS	
	Estimate	Bootstrap SE	*P* value	Estimate	SE	*P* value
Restraint	-0.003	(0.002)	0.04	-0.003	(0.002)	0.03
Hospitalization	0.010	(0.012)	0.40	0.011	(0.012)	0.36
Rehospitalization	-0.004	(0.002)	0.12	-0.004	(0.002)	0.08
Discharge	-0.002	(0.004)	0.62	-0.003	(0.004)	0.51

<u>Notes</u>: We apply both the synthetic difference-in-differences (SDID) estimator and the imputation estimator to a balanced facility-level panel. The analytic sample consists of 2,491 nursing facilities observed annually from 2017 through 2021. Columns 1-3 report SDID estimates of the association between employing a qualified social worker and each outcome, while Columns 4-6 report corresponding estimates from the imputation method. "Restraint" = The proportion of residents restrained at the annual certification survey. "Hospitalization" = The number of hospital transfers from the facility during the year divided by total resident-years. "Rehospitalization" = The proportion of new post-acute admissions from hospitals transferred to an acute hospital within 30 days. "Discharge" = The proportion of new post-acute admissions from hospitals discharged alive to the community within 100 days, without a subsequent nursing home admission within 30 days. Standard errors are obtained using bootstrap inference for SDID and cluster-robust inference at the facility level for the imputation estimates.

eMethods. Synthetic Difference-in-Differences with Staggered Adoption

Overview

We estimated the association between employment of a qualified social worker (QSW) and nursing home (NH) quality-of-care outcomes using synthetic difference-in-differences (SDID) with staggered adoption.[1,2] SDID combines key features of traditional difference-in-differences (DID) and synthetic control (SC) methods and has been increasingly applied in health and policy evaluation.[3,4] Like DID, SDID estimates differential changes in outcomes before and after treatment; like SC, it reweights control units to better match the pre-treatment trends of treated units.

Data structure and treatment definition

Let $i = 1, \ldots, N$ index NH facilities and $t = 1, \ldots, T$ index calendar years. Let $Y_{i,t}$ denote an outcome for facility i in year t. Let $D_{i,t}$ be an indicator for QSW employment at facility i in year t, taking the value 1 if a QSW is employed and 0 otherwise. Treatment adoption was staggered: facilities first employed a QSW between 2018 and 2021 and remained treated thereafter. Facilities that never employed a QSW between 2017 and 2021 served as the donor pool for constructing the synthetic comparison group. Let a_i denote the first year of QSW employment for adopting facility i, such that $D_{i,t} = 0$ for $t < a_i$ and $D_{i,t} = 1$ for $t \geq a_i$.

Estimand

The estimand of interest is the average treatment effect on the treated (ATT) across post-adoption periods among facilities that adopt QSW employment. With staggered adoption, this estimand is defined as a weighted average of cohort-specific SDID treatment effects, where cohorts are defined by the year of adoption.

Cohort-specific SDID estimation

© 2026 Chen Y et al. *JAMA Network Open.*

SDID with staggered adoption is implemented by estimating cohort-specific SDID models. For each adoption year $a \in \mathcal{A}$, where $\mathcal{A}$ denotes the set of unique adoption years, we proceeded as follows.

1. We restricted the sample to facilities that first employed a QSW in year a and to facilities that had not yet employed a QSW by year a, including never-treated facilities; the latter served as donor facilities. We refer to this subsample $\delta^{(a)}$ as the analysis sample in the adoption cohort a.
2. For each cohort a, SDID estimates the treatment effect $\hat{\tau}^{(a)}$ by solving the following regularized weighted least squares problem:

$$\left(\hat{\tau}^{(a)}, \hat{\mu}^{(a)}, \widehat{\alpha_i}^{(a)}, \widehat{\gamma_t}^{(a)}\right)$$

$$= \underset{\tau,\mu,\alpha,\gamma}{\arg\min} \sum_{i\in\delta^{(a)}} \sum_{t=1}^{T} \widehat{\omega}_i^{(a)} \hat{\lambda}_t^{(a)} \left(Y_{i,t} - \mu - \alpha_i - \gamma_t - \tau D_{i,t}\right)^2 + R\left(\omega^{(a)}, \lambda^{(a)}\right),$$

$$\text{subject to } \textstyle\sum_{i\in\delta^{(a)}} \widehat{\omega}_i^{(a)} = 1, \widehat{\omega}_i^{(a)} \geq 0, \sum_{t<a} \hat{\lambda}_t^{(a)} = 1, \hat{\lambda}_t^{(a)} \geq 0,$$

where μ is an intercept, α_i are facility fixed effects, γ_t are year fixed effects, $\omega_i^{(a)}$ are unit weights for donor facilities, $\lambda_t^{(a)}$ are time weights for pre-treatment years. Facility fixed effects (α_i) capture time-invariant facility characteristics as well as time-invariant county- and state-level characteristics, while year fixed effects (γ_t) capture common temporal shocks affecting all facilities, including nationwide changes during the COVID-19 pandemic. We did not include additional covariates in the main specification.

The regularization term $R(\cdot)$ penalizes extreme or unstable unit weights and ensures uniqueness and numerical stability of the solution. Specifically, it shrinks the unit weights toward a more dispersed distribution, preventing overfitting to pre-treatment noise while preserving close alignment of pre-treatment outcome trajectories. Time weights are

constrained to emphasize pre-treatment periods that best predict post-treatment outcomes for donor facilities. The unit and time weights are therefore chosen to minimize imbalance in pre-treatment outcomes between treated facilities and a weighted combination of donor facilities, improving pre-treatment fit and stabilizing the pre–post comparison.

Aggregation across cohorts

The overall ATT is computed as a weighted average of cohort-specific effects:

$$\widehat{ATT} = \sum_{a \in \mathcal{A}} \frac{T_{post}^{(a)}}{T_{post}} \times \hat{\tau}^{(a)},$$

where T_{post} is the total number of post-treatment years observed in treated facilities and $T_{post}^{(a)}$ is the total number of post-treatment years observed in treated facilities in cohort a.

Uncertainty quantification and hypothesis testing (bootstrap)

We reported bootstrap standard errors based on 500 repetitions for SDID estimates. The bootstrap resampled facilities (clusters) with replacement to approximate the sampling distribution while preserving within-facility serial correlation. For each bootstrap replicate, SDID weights and cohort-specific effects were re-estimated prior to aggregating the overall ATT. The bootstrap standard error was calculated as the standard deviation of the replicate ATT estimates. All tests were two-sided. Statistical significance was defined as *P*-value < 0.05.

Identification assumptions and interpretation

SDID is a design-based approach that estimates an average post-treatment contrast between treated facilities and a weighted synthetic control constructed from the donor pool. In this study, we interpret SDID estimates as associations, not causal effects. Interpretation of the SDID estimate as a causal effect would require the following conditions:

© 2026 Chen Y et al. *JAMA Network Open.*

1. **No interference (stable units).** Outcomes for a facility are not affected by QSW adoption at other facilities (no spillovers), and each facility's outcome is well-defined under treated versus untreated status.
2. **Consistency and no anticipation.** Observed outcomes equal the potential outcome under the facility's realized treatment status, and treatment does not affect outcomes prior to adoption (no anticipatory effects).
3. **Comparability after weighting (weighted parallel trends).** After applying SDID unit and time weights to achieve close pre-treatment alignment, treated facilities and their weighted synthetic controls would have continued to follow similar outcome trajectories in the absence of treatment, apart from common shocks captured by year fixed effects. Because the impacts of COVID-19 may have varied across geography and over time, year fixed effects may not absorb all differential shocks. We therefore assess robustness by augmenting the SDID specification with measures of local COVID-19 severity (annual average daily state-level COVID-19 confirmed cases and deaths).
4. **Sufficient donor support.** The donor pool contains facilities whose pre-treatment outcome patterns can adequately reproduce the treated group's pre-treatment trajectory (i.e., the treated trajectory lies within the support of the donor pool).
5. **Stable measurement over time.** Outcome definitions and reporting are comparable over time; if reporting practices change differentially with adoption, estimates may be biased. We therefore assessed pre-treatment trends using event-study analyses.
6. **Correct uncertainty quantification under clustering.** Bootstrap resampling at the facility level provides an appropriate approximation to the sampling distribution when outcomes are serially correlated within facilities.

© 2026 Chen Y et al. *JAMA Network Open.*

eReferences.

1. Arkhangelsky D, Athey S, Hirshberg DA, Imbens GW, Wager S. Synthetic difference-in-differences. *American Economic Review*. 2021;111(12):4088-4118. doi:10.1257/aer.20190159
2. Clarke D, Pailañir D, Athey S, Imbens G. On synthetic difference-in-differences and related estimation methods in stata. *The Stata Journal: Promoting communications on statistics and Stata*. 2024;24(4):557-598. doi:10.1177/1536867x241297914
3. Kakara M, Bair EF, Venkataramani AS. Repeal of Subminimum Wages and Social Determinants of Health Among People With Disabilities. *JAMA Health Forum*. 2024;5(11):e244034. Published 2024 Nov 1. doi:10.1001/jamahealthforum.2024.4034
4. Nguyen HV, Mital S, Bugden S, McGinty EE. Safer Opioid Supply, Subsequent Drug Decriminalization, and Opioid Overdoses. *JAMA Health Forum*. 2025;6(3):e250101. Published 2025 Mar 7. doi:10.1001/jamahealthforum.2025.0101

© 2026 Chen Y et al. *JAMA Network Open.*
