# Supplementary material for: Staffing of Qualified Social Workers and Nursing Home Quality of Care
Source: JAMA Netw Open. 2026 Feb 25;9(2):e260074. doi: 10.1001/jamanetworkopen.2026.0074 (PMC12936878; doi:10.1001/jamanetworkopen.2026.0074)
Supplement: Supplement 2. — Data Sharing Statement [file jamanetwopen-e260074-s002.pdf]

Data Sharing Statement

Chen. Staffing of Qualified Social Workers and Nursing Home Quality of Care. *JAMA Netw Open*. Published February 25, 2026. doi:10.1001/jamanetworkopen.2026.0074

Data
Data available: No

Additional Information
Explanation for why data not available: The data we used are publicly available but we do not have the right to redistribute the data.
